# Supplementary material for: Utility of adding Radiomics to clinical features in predicting the outcomes of radiotherapy for head and neck cancer using machine learning
Source: PLoS One. 2022 Dec 15;17(12):e0277168. doi: 10.1371/journal.pone.0277168 (PMC9754241; doi:10.1371/journal.pone.0277168)
Supplement: S1 Checklist — (PDF) [file pone.0277168.s001.pdf]

Appendix

S1A. Radiomics Quality Score: (https://www.radiomics.world/rqs)

1. Image protocol quality - well-documented image protocols (for example, contrast, slice thickness, energy, etc.) and/or usage of public image protocols allow reproducibility/replicability
    - Protocols well documented
2. Multiple segmentations - possible actions are: segmentation by different physicians/algorithms/software, perturbing segmentations by (random) noise, segmentation at different breathing cycles. Analyse feature robustness to segmentation variabilities
    - No
3. Phantom study on all scanners - detect inter-scanner differences and vendor-dependent features. Analyse feature robustness to these sources of variability
    - No/Not Applicable
4. Imaging at multiple time points - collect images of individuals at additional time points. Analyse feature robustness to temporal variabilities (for example, organ movement, organ expansion/shrinkage)
    - No/Not Applicable
5. Feature reduction or adjustment for multiple testing - decreases the risk of overfitting. Overfitting is inevitable if the number of features exceeds the number of samples. Consider feature robustness when selecting features
    - Either measure is implemented
6. Multivariable analysis with non Radiomics features (for example, EGFR mutation) - is expected to provide a more holistic model. Permits correlating/inferencing between Radiomics and non Radiomics features.
    - Yes
7. Detect and discuss biological correlates - demonstration of phenotypic differences (possibly associated with underlying gene–protein expression patterns) deepens understanding of Radiomics and biology.
    - No/Not Applicable
8. Cut-off analyses - determine risk groups by either the median, a previously published cut-off or report a continuous risk variable. Reduces the risk of reporting overly optimistic results.
    - Yes
9. Discrimination statistics - report discrimination statistics (for example, C-statistic, ROC curve, AUC) and their statistical significance (for example, p-values, confidence intervals). One can also apply resampling method (for example, bootstrapping, cross-validation)
    - a discrimination statistic and its statistical significance are reported
    - a resampling method technique is also applied

10. Calibration statistics - report calibration statistics (for example, Calibration-in-the-large/slope, calibration plots) and their statistical significance (for example, P-values, confidence intervals). One can also apply resampling method (for example, bootstrapping, cross-validation)
    - None
11. Prospective study registered in a trial database - provides the highest level of evidence supporting the clinical validity and usefulness of the Radiomics biomarker.
    - No/ Not Applicable
12. Validation - the validation is performed without retraining and without adaptation of the cut-off value, provides crucial information with regard to credible clinical performance.
    - validation is based on a dataset from the same institute
13. Comparison to 'gold standard' - assess the extent to which the model agrees with/is superior to the current 'gold standard' method (for example, TNM-staging for survival prediction). This comparison shows the added value of Radiomics.
    - Yes
14. Potential clinical utility - report on the current and potential application of the model in a clinical setting (for example, decision curve analysis).
    - Yes
15. Cost-effectiveness analysis - report on the cost-effectiveness of the clinical application (for example, QALYs generated)
    - No/ Not Applicable
16. Open science and data - make code and data publicly available. Open science facilitates knowledge transfer and reproducibility of the study.
    - the code is open sourced
    - Radiomics features are calculated on a set of representative ROIs and the calculated features and representative ROIs are open source

Total Score 16 (44.44%)

S1B. Sample Size Calculation

The sample size was calculated for this study using Relative Hazards [24]. Its computation is illustrated as follows:

$$\boldsymbol{n} = \frac{(Z_{1-\alpha/2}+Z_{1-\beta})^2}{((\log(RH))^2)(q_0 q_1)} \quad \text{.......(1)}$$

Here:

Threshold for rejecting null hypothesis is Type 1 error rate

$Z_{1-\alpha/2}$ **= 1.96** for Z value at 95% confidence level with α=0.05

Probability of failing to reject the null hypothesis under the alternate hypothesis Type II error rate

$\boldsymbol{Z_{1-\beta}}$ **= 0.84** for Z value at 80% power with β=0.80

$\boldsymbol{q_0}$ = proportion of patients had recurrence = 7 (calculated from pilot study of total 21 samples)

$\boldsymbol{q_1 = 1 - q_0}$

RH= Relative Hazard ratio (collected from previous study [25]) =1.450

On substituting these values in equation (1) the n value is calculated as:

n= 256

S1C. Calculations for performance metrics[26]

- Confusion Matrix
  The confusion matrix is calculated by comparing the classifier's predicted output with the actual outcomes. The components of confusion matrix are illustrated as follows:

		Actual Values	
		Positive (1)	Negative (0)
Predicted Values	Positive (1)	True Positive(TP)	False Positives (FP)
	Negative (0)	False Negatives (FN)	True Negatives

- Mean Accuracy

$$\sum_{i=1}^{N}\left(\frac{TP + TN}{TP + TN + FP + FN}\right)/N$$

  Here N= Number of iterations

- Mean Sensitivity

$$\sum_{i=1}^{N}\left(\frac{TP}{TP + FN}\right)/N$$

- Mean Specificity

$$\sum_{i=1}^{N}\left(\frac{TN}{TN + FP}\right)/N$$

- Mean Precision

$$\sum_{i=1}^{N}\left(\frac{TP}{TP + FP}\right)/N$$

- Recall

$$\sum_{i=1}^{N}(\frac{TP}{TP+FN})/N$$

- F1 Score

$$\frac{2\times(precision\times recall)}{precision+recall}$$

- Macro F1 Score

  It is the arithmetic mean of per- class F1 scores. In our case we have binary classification outcome. Hence Macro F1 Score is calculated as follows:

$$\frac{F1\ score\ for\ class\ label\ 0+F1\ score\ for\ class\ label\ 1}{2}$$

- Weighted F1 Score
  It will compute weighted F1 score, by considering weights of individual classes. It is calculated as follows:

$$\frac{((w0\times F1\ score(class\ 0))+(w1\times F1\ score(class\ 1)))}{Total\ number\ of\ samples}$$

  Here:

  w0= number of samples belonging to class 0

  w1= number of samples belonging to class 1
