# Supplementary material for: Utility of adding Radiomics to clinical features in predicting the outcomes of radiotherapy for head and neck cancer using machine learning
Source: PLoS One. 2022 Dec 15;17(12):e0277168. doi: 10.1371/journal.pone.0277168 (PMC9754241; doi:10.1371/journal.pone.0277168)
Supplement: S1 Appendix — (PDF) [file pone.0277168.s002.pdf]

Protocol (Description of the Project)

1. Title of the project: Development of an efficient data analytics model for Head and Neck Cancer using Clinical features and Radiomics.

2. Type of Study: Retrospective

3. Aims & objectives (hypotheses if applicable):

a. To understand the patterns of care, trends of treatment and treatment/follow-up compliance for patients with head & neck squamous cell cancers at Kasturba Hospital, Manipal.

b. To identify new prognostic factors based on patient, imaging and treatment characteristics by comparing them with disease outcomes.

c. To identify prognostic and predictive factors from qualitative and quantitative analysis of cross-sectional images (CT/MRI) of the patients and comparing them with treatment outcomes.

4. Justification for study (whether of national significance with rationale): The research challenge lies in identifying the trends of cancer treatment effectiveness based on the imaging and non-imaging data of the patient along with his/her medical information. Subsequently, this entire data can be used for classifying the patients based on the cancer stage and treatment response. The challenge also lies in understanding the various costs incurred by the Hospital and the patient and identify ways to optimize the same. This model should be cost effective, and should determine the factors of the state of wellness of the patient, such as survival rate, probability of recurrence of disease and will provide information about the effectiveness of the treatment.

5. Departments involved: Department of Radiotherapy and Oncology, Department of Radiology, School of Information Science

6. Study period: 3 Years and 6 Months

7. Sample size : All the patients with Head and Neck cancer treated at our centre in between years 2013 and 2018.

Sample Size Calculation

Sample size calculated based on Relative Hazard as shown in equation 1

$$n= \frac{(Z_{1-\alpha/2}+Z_{1-\beta})^2}{\log(RH)^2\ (q_0\ X\ q_1)} \qquad 1)$$

Where

$Z_{1-\alpha/2}$= z value at 95 % confidence level (α=0.05). This is the thresholding probability for rejecting the Null Hypothesis. *Type 1 error rate.*

=**1.96**

$Z_{1-\beta}$= z value at 80 % power (β=0.80). This is the probability of failing to reject the null hypothesis under the alternative hypothesis. *Type II error rate*.
= **0.84**

q_0= Proportion of patient had recurrence for Head and Neck Cancer = **0.333**

q_1= 1- q_0

RH= Relative hazard Ratio = **1.450** (Collected from previous studies) [4]

From the equation 1, n value is calculated and results obtained are as follows:
n= 256

8. Materials and methods:

a) Inclusion and exclusion criteria:

Inclusion:

1. Patients with head and neck squamous cell carcinomas who underwent treatment at Kasturba Hospital, Manipal.

Exclusion:

1. Patients who were unwilling for any further evaluation or further treatment, and those who had visited the center for a 2nd opinion.
2. Patients who are lost to follow up following treatment completion, or have less than 3 months' follow up.

b) Biological materials required (type - blood, tissue etc and quantity): NA

c) Statistical methods: NA

9. Detailed description of procedure / processes:

Description of Pilot Study:

1. Clinical data collection from the patient medical reports including histopathology reports, identifying all the relevant parameters and checking its availability and feasibility for multiple patients (dataset will be anonymised before collecting).
2. Checking for the missing data and using suitable methodology for filling the data (statistically or analytically).
3. Imaging data collection from the radiological images, annotating the ROI and extracting radiomic features with the help of clinical expertise (without using CAD tools for image segmentation or feature extraction).
4. Statistically finding the critical parameters and determining the significant features for justifying which features should be used for the overall model creation.

5. Finding the research gaps identified from pilot study.

Description of complete research (After Pilot Study):

Step 1

- Collection of larger dataset, for the critical parameters justified in pilot study.
- Detection of outliers for each selected parameter, to be included for the development of data analytics model.

Step 2

- Collection of larger dataset of Radio diagnosis images.
- Implementing CAD tools for segmentation and feature extraction from ROI from the images.
- Comparing the extracted ROI from the clinical annotations, with the one segmented out from the automated segmentation.
- From the extracted ROI, extract the features with the help of a suitable algorithm.
- Comparing the obtained radiomics features (From the algorithm) with the critical features obtained from the pilot study (from the expertise of clinicians) and justifying the critical features for large dataset.

Step 3:

- Combining all the features obtained from phase 1 and 2 and it will work as input to the data analytics algorithm.
- Diving the dataset appropriately according to the training set, validation set and the test set.
- Development of the algorithm using machine learning tool, for the classification according to the staging of cancer and clustering according to the highly correlated parameters.
- Comparing the diagnostic accuracy and performance of the algorithms with others, and finding the best data model for the dataset and validating with new dataset.

10. Outcome measures: The outcome of this project will provide a data analytics model which will input the clinical, radiomic and pathomic parameters of the patient, and give the critical features for better prognosis. This model will also determine the stage of the cancer, with respect to the critical parameters identified in the research. This will also determine the effect of treatment on the patient, possibility of reoccurrence, Cost effectiveness, survival rate etc.

11. Potential risks and benefits : The study looks retrospectively into the anonymized patient details, and therefore carries neither any risk nor any benefit to the patient who has been included into the analysis.

12. Ethical considerations and methods to address issues : There are no recognized ethical concerns with the conduct of this study. All patient data including imaging data will be anonymized.

13. Budget (give details) and proposed funding source: NA

14. Review of literature (within 1000 words): In the year 2014, Chintan Parmar et al, worked on robust radiomic feature quantification using semiautomatic volumetric segmentation. In this study, a semiautomatic region growing volumetric segmentation algorithm, implemented in the free and publicly available 3D-Slicer platform, was investigated in terms of its robustness for quantitative imaging feature extraction. Fifty-six 3D-radiomic features, quantifying phenotypic differences based on tumor intensity, shape and texture, were extracted from the computed tomography images of twenty lung cancer patients. These radiomic features were derived from the 3D-tumor volumes defined by three independent observers twice using 3D-Slicer, and compared to manual slice-by-slice delineations of five independent physicians in terms of intra-class correlation coefficient (ICC) and feature range. Radiomic features extracted from 3D-Slicer segmentations had significantly higher reproducibility (ICC = 0.8560.15, p = 0.0009) compared to the features extracted from the manual segmentations (ICC = 0.7760.17). Furthermore, it was found that features extracted from 3D-Slicer segmentations were more robust, as the range was significantly smaller across observers (p = 3.819e-07), and overlapping with the feature ranges extracted from manual contouring (boundary lower: p = 0.007, higher: p = 5.863e-06). Our results show that 3D-Slicer segmented tumor volumes provide a better alternative to the manual delineation for feature quantification, as they yield more reproducible imaging descriptors. Therefore, 3D-Slicer can be employed for quantitative image feature extraction and image data mining research in large patient cohorts.

In the year 2017, Martin Vallieres et al, worked on Radiomic strategies for risk assessment of tumour failure in Head and Neck Cancer. In this study, 1615 radiomic features (quantifying tumour image intensity, shape, texture) extracted from pre- treatment FDG-PET and CT images of 300 patients from four different cohorts were analysed for the risk assessment of loco regional recurrences (LR) and distant metastases (DM) in head-and-neck cancer. Prediction models combining radiomic and clinical variables were constructed via random forests and imbalance-adjustment strategies using two of the four cohorts. Independent validation of the prediction and prognostic performance of the models was carried out on the other two cohorts (LR: AUC = 0.69 and CI = 0.67; DM: AUC = 0.86 and CI = 0.88). Furthermore, the results obtained via Kaplan-Meier analysis demonstrated the potential of radiomics for assessing the risk of specific tumour out- comes using multiple stratification groups. This could have important clinical impact, notably by allowing for a better personalization of chemo- radiation treatments for head-and-neck cancer patients from different risk groups.

In the year 2016, Andrew J Wong et al, worked on Radiomics in Head and Neck cancer from exploration to application. In this study, the radio diagnostic CT images were acquired, having

head and neck cancer. Pre-processing and segmentation is done on these images to extract out the region of interest, having high possibility of presence of Tumour. From the extracted tumours, radiomic features are extracted. The selected features can then be analysed for outcome correlation and potential incorporation into predictive models. Additionally, validations should be done against completely independent large datasets.

In the year 2015, Ralph T H Leijenaar et al worked on External validation of a prognostic CT- based radiomic signature in oropharyngeal squamous cell carcinoma. A total of 542 OPSCC patients were included for which we determined the prognostic index (PI) of the radiomic signature. We tested the signature model fit in a Cox regression and assessed model discrimination with Harrell's c-index. Kaplan-Meier survival curves between high and low signature predictions were compared with a log-rank test. Validation was performed in the complete cohort (PMH1) and in the subset of patients without (PMH2) and with (PMH3) visible CT artifacts within the delineated tumor region. It was identified 267 (49%) patients without and 275 (51%) with visible CT artifacts. The calibration slope (β) on the PI in a Cox proportional hazards model was 1.27 ($H0$: β= 1, p=0.152) in the PMH1 (n= 542), 0.855 ($H0$: β= 1,p=0.524) in the PMH2 (n= 267) and 1.99 ($H0$: β= 1, p=0.002) in the PMH3 (n =275) cohort. Harrell's c-index was 0.628 (p=2.72*e*-9), 0.634 (p=2.7*e*-6) and 0.647 (p=5.35*e*-6) for the PMH1, PMH2 and PMH3 cohort, respectively. Kaplan-Meier survival curves were significantly different ($p < 0.05$) between high and low radiomic signature model predictions for all cohorts. Overall, the signature validated well using all CT images as-is, demonstrating a good model fit and preservation of discrimination. Even though CT artifacts were shown to be of influence, the signature had significant prognostic power regardless if patients with CT artifacts were included.

In 2014, Hugo J.W.L worked on Decoding tumour phenotype by non-invasive imaging using a quantitative radiomics approach. In this study a radiomic analysis of 440 features is presented, quantifying tumour image intensity, shape and texture, which are extracted from computed tomography data of 1,019 patients with lung or head-and-neck cancer. We find that a large number of radiomic features have prognostic power in independent data sets of lung and head-and-neck cancer patients, many of which were not identified as significant before. Radio genomics analysis reveals that a prognostic radiomic signature, capturing intratumour heterogeneity, is associated with underlying gene-expression patterns. These data suggest that radiomics identifies a general prognostic phenotype existing in both lung and head-and-neck cancer. This may have a clinical impact as imaging is routinely used in clinical practice, providing an unprecedented opportunity to improve decision-support in cancer treatment at low cost.

In 2017, Philippe Lambin et al worked on Radiomics: the bridge between medical imaging and personalised medicine. Radiomic analysis exploits sophisticated image analysis tools and the rapid development and validation of medical imaging data that uses image-based signatures for precision diagnosis and treatment, providing a powerful tool in modern medicine. Herein, the process of radiomics, its pitfalls, challenges, opportunities, and its capacity to improve clinical decision making, emphasizing the utility for patients with cancer is described. Currently, the field of radiomics lacks standardized evaluation of both the scientific integrity and the clinical relevance of the numerous published radiomics investigations resulting from the rapid growth of this area.

15. References :

[1] H. J. W. L. Aerts *et al.*, “Decoding tumour phenotype by noninvasive imaging using a quantitative radiomics approach,” *Nat. Commun.*, vol. 5, 2014.

[2] C. Parmar *et al.*, “Robust radiomics feature quantification using semiautomatic volumetric segmentation,” *PLoS One*, vol. 9, no. 7, pp. 1–8, 2014.

[3] M. Vallières *et al.*, “Radiomics strategies for risk assessment of tumour failure in head-and-neck cancer,” *Sci. Rep.*, vol. 7, no. 1, pp. 1–33, 2017.

[4] Zhai TT, van Dijk L V., Huang BT, et al. Improving the prediction of overall survival for head and neck cancer patients using image biomarkers in combination with clinical parameters. *Radiother Oncol*. 2017;124(2):256-262. doi:10.1016/j.radonc.2017.07.013

[5] P. Lambin *et al.*, “Radiomics: The bridge between medical imaging and personalized medicine,” *Nat. Rev. Clin. Oncol.*, vol. 14, no. 12, pp. 749–762, 2017.

[6] R. T. H. Leijenaar *et al.*, “External validation of a prognostic CT-based radiomic signature in oropharyngeal squamous cell carcinoma,” *Acta Oncol. (Madr).*, vol. 54, no. 9, pp. 1423–1429, 2015.

[7] A. J. Wong, A. Kanwar, A. S. Mohamed, and C. D. Fuller, “Radiomics in head and neck cancer: from exploration to application,” *Transl. Cancer Res.*, vol. 5, no. 4, pp. 371–382, 2016.
