# Supplementary material for: Utility of adding Radiomics to clinical features in predicting the outcomes of radiotherapy for head and neck cancer using machine learning
Source: PLoS One. 2022 Dec 15;17(12):e0277168. doi: 10.1371/journal.pone.0277168 (PMC9754241; doi:10.1371/journal.pone.0277168)
Supplement: Attachment — Submitted filename: Response to reviewers.docx [file pone.0277168.s004.docx]

| **Date:** | 16^th^ August 2022 |
| --- | --- |
| **Subject:** | Response to the reviewers’ queries and suggestions [PONE-D-22-16089] |

(PONE-D-22-16089: Utility of adding Radiomics to clinical features in predicting outcomes of radiotherapy for Head and Neck Cancer using Machine Learning)

The authors would like to express their profuse gratitude for the reviewers’ time and efforts, and for the excellent criticisms raised in order to facilitate the betterment of our article. The queries have been addressed in the manuscript, and the replies are stated below:

Queries summarized (editor):

1) There are some ethical concerns, as you state that no consent was acquired from the participants for use of the data because "the study was retrospective"

Ethical clearance for this study was obtained from our Institutional Ethics Committee (approval no. 165/2018). Since the study involved retrospective data retrieved from medical records/imaging archives, and the patients were not contacted, participation consent couldn’t be taken, and was waived off by the IEC.

2) The methodology used is not described sufficiently

The methodology section has been updated to provide greater details in the original manuscript. (section 2.3.1-2.3.6)(page-3-6)

3) There is no split in training and validation set and maybe an additional cohort should be analysed.

Multiple iterations of Training and testing splits at 70:30 ratio was performed within the collected dataset. The details have been added and clarified in the revised manuscript (section 2.3.3-2.3.5)(page 5-6)

4) The english language used is unacceptable and the manuscript should be edited by a native speaker/ scientific writer.

The manuscript has been extensively re-written to address the grammatical and clarity errors, and has been screened through language-analysis software (professional version of Grammarly) for inaccuracies.

**Other observations:** Your ethics statement should only appear in the Methods section of your manuscript. If your ethics statement is written in any section besides the Methods, please move it to the Methods section and delete it from any other section. Please ensure that your ethics statement is included in your manuscript, as the ethics statement entered into the online submission form will not be published alongside your manuscript.

Statement moved to the methodology section as suggested.(page 4-5)

**Other observations:** Please provide additional details regarding participant consent. In the Methods section, please ensure that you have specified (1) whether consent was informed and (2) what type you obtained (for instance, written or verbal). If your study included minors, state whether you obtained consent from parents or guardians. If the need for consent was waived by the ethics committee, please include this information.

The data being retrospective, informed consent from the patients was waived off by the Institutional Ethics Committee. Its details are mentioned in the methodology section of manuscript. (page 4-5)

**Other observations:** We note that you have indicated that data from this study are available upon request. PLOS only allows data to be available upon request if there are legal or ethical restrictions on sharing data publicly. For information on unacceptable data access restrictions, please see http://journals.plos.org/plosone/s/data-availability#loc-unacceptable-data-access-restrictions.

In your revised cover letter, please address the following prompts:

a) If there are ethical or legal restrictions on sharing a de-identified data set, please explain them in detail (e.g., data contain potentially identifying or sensitive patient information) and who has imposed them (e.g., an ethics committee). Please also provide contact information for a data access committee, ethics committee, or other institutional body to which data requests may be sent.

b) If there are no restrictions, please upload the minimal anonymized data set necessary to replicate your study findings as either Supporting Information files or to a stable, public repository and provide us with the relevant URLs, DOIs, or accession numbers. Please see http://www.bmj.com/content/340/bmj.c181.long for guidelines on how to de-identify and prepare clinical data for publication. For a list of acceptable repositories, please see http://journals.plos.org/plosone/s/data-availability#loc-recommended-repositories.

We will update your Data Availability statement on your behalf to reflect the information you provide.

The collected data is the intellectual property of Manipal Academy of Higher Education, Manipal (India), and Philips, Bangalore (India), and as per the exhibit, we are not permitted to share the collected data. Moreover, we don’t have the government regulatory body (Health Ministry Screening Committee, Indian Council of Medical Research) approval for data sharing.


**Reviewers' comments to the authors:**

Reviewer #1: 

1. I suggest that a native speaker proofreads the manuscript. There are several grammar mistakes, and the general style could be improved.

The manuscript has been extensively re-written to address the grammatical and clarity errors, and has been screened through language-analysis software (professional version of Grammarly) for inaccuracies.

2. Please check the row for age in the patient characteristics table.

The data entered was incorrect; thank you very much for bringing it to our notice. The entire table has been thoroughly reviewed and corrected.(page 6-7)

3. Even though you point to a previous study, I find the paper is lacking some high-level explanation of the methodology (how was the train/test split performed, which feature selection algorithms were employed, what are the weights in the F1-score computation, etc). Right now it is very hard for the reader to understand what methodology was used in this study.

The methodology section has been updated to describe the workflow in a more coherent manner.(page 3-6)

4. I have concerns whether the follow-up time is too little to be clinically significant.

We agree that the follow-up duration, while sufficient for reporting early response, is indeed an inadequate represent of the overall clinical picture. The same has been reiterated in the final paragraph of the discussion stating the limitations of our study.(page 13-14)

Reviewer #2: 

1) How was the annotation of the PT and the LN done? Manually? By whom? was each LN delineated separately?

The annotations of PT and LN were performed using the 3D slicer tool manually by the Radiation Oncologist. The same has been mentioned in the manuscript (page 4).

2)Please provide more details on the extraction of features:
- how many features were extracted per feature type?

We extracted features under six major domains, including shape-based (14), gray-level dependence matrix (14), gray-level cooccurence matrix (24), first-order statistics (18), gray-level run length matrix (16), gray-level size zone (16) and neighboring gray-tone difference matrix (5). The same has been mentioned in the manuscript (page 4-5).

-Which pre-processing was done?

No separate pre-processing of the images was performed; radiomics features were obtained after annotating the CT images with primary and nodal volumes with the pyradiomics toolbox (an extension provided by 3D-slicer annotation tool). Prior to subjecting the data to ML training algorithms, standard scalar was performed on all the variables.(page 5-6)

- Were cubic voxels used?

Yes. Shape-based 3D-radiomics features were used in the analysis.

- Which binning method?

No binning method used, as the structured radiomics features were directly extracted from the Pyradiomics toolbox.

3) Why did you not split the dataset into a training and validation dataset? Since this is not performed a cross-validation is needed.
The dataset was split into training and testing dataset in the ratio of (70:30). The details are illustrated in the updated methodology section (). Thereafter, cross-validation was performed on complete data for all the models using stratified K-fold cross validation (page 5-6)

4) To better address all these questions please determine the radiomics quality score:
<https://www.radiomics.world/rqs> and put your score to the manuscript including your answers as an appendix.

Thank you for this suggestion! On attempting the suggested questionnaire, the determined score was, unfortunately, only 45%. However, we would like to state here that at least six of the 16 questions (No.s 3,4,7,8,11 and 15) are not applicable to our study. (Appendix)

5) Results: With clinico-radiomic data, the mean training and testing accuracy->From Materials and Methods it is not clear that you have splitted in training and test set, please explain.
The dataset was split into training-testing ratio of 70:30, and the means of 10 such iterations were the performance was reported by evaluating the designed model using test dataset. (page 8-12)

6) All your results on accuracy, sensitivity,... need confidence intervals.

Thank you again for pointing this out! Confidence intervals values have been added to all the metrics in the original manuscript. (page 8-12)
