# Supplementary material for: Utility of adding Radiomics to clinical features in predicting the outcomes of radiotherapy for head and neck cancer using machine learning
Source: PLoS One. 2022 Dec 15;17(12):e0277168. doi: 10.1371/journal.pone.0277168 (PMC9754241; doi:10.1371/journal.pone.0277168)
Supplement: Attachment — Submitted filename: Response to Reviewers.docx [file pone.0277168.s005.docx]

| **Date:** | Oct 06 2022 11:46AM |
| --- | --- |
| **To:** | "Rajagopal Kadavigere" rajarad@gmail.com |
| **From:** | "PLOS ONE" plosone@plos.org |
| **Subject:** | PLOS ONE Decision: Revision required [PONE-D-22-16089R1] |

PONE-D-22-16089R1

Utility of adding Radiomics to clinical features in predicting outcomes of radiotherapy for Head and Neck Cancer using Machine Learning

PLOS ONE

The authors would again like to thank the reviewers profusely for critically reviewing the manuscript and highlighting the points which have helped us considerably improve the quality of the manuscript. The answers to the issues raised by the reviewers have been addressed below.

Please review your reference list to ensure that it is complete and correct. If you have cited papers that have been retracted, please include the rationale for doing so in the manuscript text, or remove these references and replace them with relevant current references. Any changes to the reference list should be mentioned in the rebuttal letter that accompanies your revised manuscript. If you need to cite a retracted article, indicate the article’s retracted status in the References list and also include a citation and full reference for the retraction notice.

The reference list is updated and checked for its correctness.

Additional Editor Comments:

Dear authors,
thank you for addressing all comments.
Please answer the minor issues raised from reviewer 1 and then the manuscript is suitable for acceptance.


[Note: HTML markup is below. Please do not edit.]

Reviewers' comments:

Reviewer's Responses to Questions

**Comments to the Author**

1. If the authors have adequately addressed your comments raised in a previous round of review and you feel that this manuscript is now acceptable for publication, you may indicate that here to bypass the “Comments to the Author” section, enter your conflict of interest statement in the “Confidential to Editor” section, and submit your "Accept" recommendation.

Reviewer #1: (No Response)

Reviewer #2: All comments have been addressed

2. Is the manuscript technically sound, and do the data support the conclusions?

The manuscript must describe a technically sound piece of scientific research with data that supports the conclusions. Experiments must have been conducted rigorously, with appropriate controls, replication, and sample sizes. The conclusions must be drawn appropriately based on the data presented.

Reviewer #1: Partly

Reviewer #2: Yes

3. Has the statistical analysis been performed appropriately and rigorously?

Reviewer #1: Yes

Reviewer #2: Yes

4. Have the authors made all data underlying the findings in their manuscript fully available?

The [PLOS Data policy](http://www.plosone.org/static/policies.action#sharing) requires authors to make all data underlying the findings described in their manuscript fully available without restriction, with rare exception (please refer to the Data Availability Statement in the manuscript PDF file). The data should be provided as part of the manuscript or its supporting information, or deposited to a public repository. For example, in addition to summary statistics, the data points behind means, medians and variance measures should be available. If there are restrictions on publicly sharing data—e.g. participant privacy or use of data from a third party—those must be specified.

Reviewer #1: No

Reviewer #2: No

5. Is the manuscript presented in an intelligible fashion and written in standard English?

PLOS ONE does not copyedit accepted manuscripts, so the language in submitted articles must be clear, correct, and unambiguous. Any typographical or grammatical errors should be corrected at revision, so please note any specific errors here.

Reviewer #1: Yes

Reviewer #2: Yes

6. Review Comments to the Author

Please use the space provided to explain your answers to the questions above. You may also include additional comments for the author, including concerns about dual publication, research ethics, or publication ethics. (Please upload your review as an attachment if it exceeds 20,000 characters)

Reviewer #1: 1. Please check capitalization rules in English and change the capitalized letters accordingly throughout the whole manuscript (e.g., in one sentence you write "artificial intelligence" and "Machine Learning").

The sentences were checked thoroughly for capitalization and corrected throughout the manuscript.

2. It is not clear what you mean by "Though the minimum sample size was estimated to be 256, we included all the eligible patients treated between 2013 -2018".

The minimum sample size was calculated for this study as 256 using the proportions of relative hazards formula. The sample size calculation is shown in section 9.2 Appendix. This was the minimum sample size required; however, we screened all the 482 records of patients treated between 2013-18 and selected all the 311 eligible patient records, because a larger sample size is expected to provide better performing models.

3. "Synthetic samples were taken out from the original samples. The training: testing split was
performed with a 70:30 ratio on the class having majority samples. The training dataset was
generated by adding the train-split of majority samples to the synthetic data, and the testing
dataset was generated by adding test-split of the majority samples to the original minority
samples".

Please rephrase this paragraph and clearly state the class imbalance on the training set and on the test set for each clinical endpoint.

The paragraph has been simplified and rephrased in the manuscript. Also, the number of samples for the training and testing dataset is specified for each clinical endpoints in table 2, 3,4 and 5.

4. Please specify the weights of the F1-score calculation and also provide the non-weighted F1-score.

In the manuscript, Tables 2,3,4 and 5 have been updated with Training and testing f1 score, macro f1 score and weighted f1 score for each class (label 0 and 1). The number of samples for Training and testing class label 0 and 1 serves as the weights to calculate weighted f1 score. The calculation for each performance metrics is presented in section 9.3 appendix.

5. The LRC model is heavily overfitted. Could you implement any measure to prevent this from happening? Such as other feature reduction techniques.

In this research we have applied intrinsic pre-processing steps to clean the dataset and balance for the class labels. In order to prevent overfitting for locoregional recurrence models, we tried the following additional steps:

1) Computed Principal Component Analysis(PCA) for the original dataset without and with feature selection using Sequential Forward Floating Selection and visualised the results for two principal components. However, a clear class boundary which separates two variables couldn’t be drawn. The PCA plots are shown as follows:

a) b)

Figure: PCA plot of two components for only clinical data a) without feature selection and b) with feature selection

a) b)

Figure: PCA plot of two components for clinico radiomics data a) without feature selection and b) with feature selection

a) b)

Figure: PCA plot of two components for only radiomics data a) without feature selection and b) with feature selection

2) Originally have used the ‘accuracy’ hyperparameter for selecting optimal features. We have redone the analysis for locoregional models by changing the hyperparameter setting to ‘f1’and ‘f1_weighted’
3) Finally, we tried to vary the learning rate ‘C’ in KSVM for Only Clinical and Clinico-Radiomics dataset and ‘max_depth’ in RF for Only Radiomics dataset. The training and testing accuracy variation with the variability of learning rate parameters is shown in the table as follows:

|  | Hyper Parameter variation | Only clinical KSVM | Clinico radiomics  KSVM |
| --- | --- | --- | --- |
| KSVM | C= 0.1 | Training Weighted F1:0.53  Testing Weighted F1: 0.33 | Training Weighted F1: 0.44  Testing Weighted F1: 0.25 |
|  | C= 0.2 | Training Weighted F1:0.69  Testing Weighted F1:0.48 | Training Weighted F1: 0.66  Testing Weighted F1: 0.41 |
|  | C= 0.3 | Training Weighted F1:0.76  Testing Weighted F1: 0.49 | Training Weighted F1: 0.86  Testing Weighted F1: 0.53 |
|  | C= 0.4 | Training Weighted F1: 0.95  Testing Weighted F1: 0.50 | Training Weighted F1: 0.99  Testing Weighted F1: 0.53 |
|  | C= 0.5 | Training Weighted F1: 0.96  Testing Weighted F1: 0.52 | Training Weighted F1:0.99  Testing Weighted F1: 0.59 |
|  | C= 0.6 | Training Weighted F1:0.96  Testing Weighted F1: 0.55 | Training Weighted F1: 0.99  Testing Weighted F1: 0.66 |
|  | C= 0.7 | Training Weighted F1: 0.97  Testing Weighted F1: 0.57 | Training Weighted F1: 1  Testing Weighted F1: 0.82 |
|  | C= 0.8 | Training Weighted F1:0.96  Testing Weighted F1: 0.73 | Training Weighted F1:1  Testing Weighted F1:0.81 |
|  | C= 0.9 | Training Weighted F1:0.97  Testing Weighted F1: 0.72 | Training Weighted F1:1  Testing Weighted F1:0.81 |
|  | C= 1.0 | Training Weighted F1:0.97  Testing Weighted F1: 0.72 | Training Weighted F1:1  Testing Weighted F1:0.81 |
|  | C= 1000 | Training Weighted F1:1  Testing Weighted F1: 0.76 | Training Weighted F1:1  Testing Weighted F1:0.78 |
| RF | max_depth=5 | Training Weighted F1:0.84  Testing Weighted F1: 0.65 | |
|  | max_depth=10 | Training Weighted F1:0.86  Testing Weighted F1: 0.68 | |
|  | max_depth=50 | Training Weighted F1:0.86  Testing Weighted F1: 0.66 | |
|  | max_depth=100 | Training Weighted F1:0.86  Testing Weighted F1: 0.64 | |
|  | max_depth=1000 | Training Weighted F1:0.85  Testing Weighted F1: 0.65 | |

Thus, we concluded that class labels have high overlap due to limitation of low number of samples, the algorithm was bound to overfit. With a greater sample size and higher numbers of positive samples, it might be feasible to prevent this.

Reviewer #2: Thanks. It is a pity that the data cannot be made fully available. Please consider for th future to apply for this. I understand this is not easy in medicine but it should still be an aim. I appriciate the calculation of radiomics quality score, It has an average score, the score is important that the reader can easily understand if the manuscript is more hypothesis generating or statistically significant.

7. PLOS authors have the option to publish the peer review history of their article ([what does this mean?](https://journals.plos.org/plosone/s/editorial-and-peer-review-process#loc-peer-review-history)). If published, this will include your full peer review and any attached files.


If you choose “no”, your identity will remain anonymous but your review may still be made public.


**Do you want your identity to be public for this peer review?** For information about this choice, including consent withdrawal, please see our [Privacy Policy](https://www.plos.org/privacy-policy).

Reviewer #1: No

Reviewer #2: No
